# Serine protease dynamics revealed by NMR analysis of the thrombin-thrombomodulin complex

**DOI:** 10.1038/s41598-021-88432-z

**Published:** 2021-04-30

**Authors:** Riley B. Peacock, Taylor McGrann, Marco Tonelli, Elizabeth A. Komives

**Affiliations:** 1 Department of Chemistry and Biochemistry, University of California San Diego, 9500 Gilman Drive, La Jolla, CA 92093-0378 USA; 2 NMRFAM University of Wisconsin, 433 Babcock Drive, Madison, WI 53706 USA

**Keywords:** Biochemistry, Biophysics

## Abstract

Serine proteases catalyze a multi-step covalent catalytic mechanism of peptide bond cleavage. It has long been assumed that serine proteases including thrombin carry-out catalysis without significant conformational rearrangement of their stable two-β-barrel structure. We present nuclear magnetic resonance (NMR) and hydrogen deuterium exchange mass spectrometry (HDX-MS) experiments on the thrombin-thrombomodulin (TM) complex. Thrombin promotes procoagulative fibrinogen cleavage when fibrinogen engages both the anion binding exosite 1 (ABE1) and the active site. It is thought that TM promotes cleavage of protein C by engaging ABE1 in a similar manner as fibrinogen. Thus, the thrombin-TM complex may represent the catalytically active, ABE1-engaged thrombin. Compared to apo- and active site inhibited-thrombin, we show that thrombin-TM has reduced μs-ms dynamics in the substrate binding (S1) pocket consistent with its known acceleration of protein C binding. Thrombin-TM has increased μs-ms dynamics in a β-strand connecting the TM binding site to the catalytic aspartate. Finally, thrombin-TM had doublet peaks indicative of dynamics that are slow on the NMR timescale in residues along the interface between the two β-barrels. Such dynamics may be responsible for facilitating the N-terminal product release and water molecule entry that are required for hydrolysis of the acyl-enzyme intermediate.

## Introduction

Serine proteases have provided textbook examples of enzyme catalysis since the first crystal structure of chymotrypsin was solved in 1967^1^. The well-known catalytic triad, the S1 substrate-binding pocket, and the oxy-anion hole provide nucleophilic and acid–base catalysis. The myriad of serine protease crystal structures show that the arrangement of these critical structural elements is highly similar leading to the assumption that serine proteases transition between their catalytic steps without significant structural rearrangements. We have been studying the serine protease, thrombin, which catalyzes the last step of the blood-clotting protease cascade. The crystal structure of α-thrombin reveals the canonical chymotrypsin (CT) fold, comprised of an N-terminal and a C-terminal β-barrel, as well as an aligned catalytic triad containing His 57_CT_ (79_seq_), Asp 102_CT_ (135_seq_), and Ser 195_CT_ (241_seq_)^2^. It should be noted that the thrombin residues numbered according to the chymotrypsin system will be referenced with a “CT” subscript, while those numbered using the sequential system will include “seq”. The proteolytic cleavage of the zymogen, prothrombin, generates the new N-terminus of the thrombin heavy chain, which is observed to be inserted into the Ile cleft forming a key interaction with Asp 194 and positioning Ser 195 for catalysis^3–5^. Using hydrogen–deuterium exchange mass spectrometry (HDX-MS), we previously showed, however, that the new N-terminal peptide is highly exchanging in the absence of the covalent active site inhibitor D-Phe-Pro-Arg-chloromethylketone (PPACK)^6^. In fact, binding of PPACK reduced exchange throughout the thrombin molecule. Similarly, NMR experiments comparing apo- and PPACK-thrombin showed that the presence of PPACK leads to the appearance of many, strong resonances that are either weak or absent in the apo-thrombin spectrum^7,8^. A number of thrombin resonances remained absent from heteronuclear single quantum coherence (HSQC) spectra of PPACK thrombin in multiple NMR experiments^9,10^. These missing resonances were largely localized to the loop regions of thrombin, which are the main structural elements that distinguish thrombin from trypsin and chymotrypsin, suggesting that the thrombin loops remain dynamic even when an inhibitor is bound at the active site. HDX-MS and accelerated molecular dynamics (AMD) simulations also reported the loops of thrombin to be conformationally dynamic^6–8,11^. In addition, Carr-Purcell-Meiboom-Gill (CPMG) experiments revealed a large number of backbone NH groups undergo μs-ms dynamics throughout apo thrombin and a pathway of residues connecting the allosteric site (ABE1) to the active site remain mobile in PPACK thrombin^8^.

The loops of thrombin have been shown to play important roles in thrombin activity. Residues from the 170s_CT_, 180s_CT_, and 220s_CT_ loops have been implicated in the Na^+^-mediated allosteric activation of thrombin through the structuring of the S1 binding site that results from the presence of a Na^+^ ion bound in this region^12,13^. Residues within the 30s_CT_ and 70s_CT_ loops interact with the thrombin cofactor thrombomodulin (TM). TM binding toggles the substrate specificity of thrombin away from pro-coagulative substrates that encourage clotting once proteolytically cleaved by thrombin, and towards the anticoagulative thrombin substrate protein C, which acts to shut down the clotting cascade once activated by thrombin^14^. Though TM contains multiple domains, the TM456 fragment containing only the 4th, 5th, and 6th EGF-like domains of TM is able to invoke the full anticoagulative activity of thrombin^15,16^. The active site inhibitor, D-Glu-Gly-Arg-chloromethylketone (GGACK), was required to solve the crystal structure of thrombin bound to TM456^17^. Surface plasmon resonance reported rapid association and dissociation rates (6.7 × 10^6^ M^-1^ s^-1^ and 0.033 s^-1^ respectively) for the interaction of TM456 with thrombin^18^, and Isothermal titration calorimetry (ITC) showed that TM456-binding is primarily driven by a change in entropy rather than enthalpy^19,20^. HDX-MS experiments on thrombin in the presence and absence of TM have reported reductions in amide exchange in regions of thrombin –such as the active site- that are distant from the TM-binding site, consistent with the fact that TM is an allosteric modulator of thrombin function^6,11^.

Although multiple studies have investigated the impact of TM binding on thrombin dynamics, there is still no clear explanation as to how TM binding modulates the catalytic activity of the protease. Here we report NMR CPMG experiments to characterize the dynamic consequences of TM456 binding to thrombin on the µs-ms timescale^21,22^. Complementary use of HDX-MS reveals changes in the dynamics of some of the loops for which NMR resonances were missing^23,24^. Through the complementary use of these two biophysical methods, we report the identities of the thrombin residues that facilitate allosteric communication between the TM binding site, the catalytic residues, and the S1 binding pocket. TM binding remodels thrombin dynamics on multiple timescales; ordering the substrate binding pocket while also promoting the conformational flexibility required for the mechanistic steps of the serine protease reaction.

## Results

### Resonance assignments and chemical shift perturbations

We previously reported resonance assignments, backbone dynamics and CPMG analysis of the μs-ms dynamics for apo thrombin^8^ and PPACK thrombin^8,10^. In this previous work, we were able to assign 82% of the thrombin sequence in PPACK-thrombin and 73% in apo-thrombin. Here we have analyzed the complex between apo thrombin and the fragment of thrombomodulin, TM456, which is fully active in promoting protein C cleavage/activation^6^. The sample used for NMR experimentation contained a 1.5 molar excess of unlabeled TM to labeled thrombin. The thrombin-TM456 spectra more closely resembled the apo-thrombin and a similar percentage of amino acids could be assigned (Supplementary Fig. 1). Every single cross peak in the spectra was assigned suggesting that missing cross peaks are likely due to conformational heterogeneity. Nine residues- clustered around the C-terminal $\beta$-barrel and the S1 pocket- which were missing from the apo-thrombin spectra could be assigned in thrombin-TM456. These nine residues were also observed in PPACK-thrombin HSQC spectra. Interestingly, four assignments were present in the apo-thrombin spectrum that were missing from the thrombin-TM456 spectra, and these were all located within the N-terminal $\beta$-barrel.

There were 23 residues in the thrombin-TM456 spectra that showed multiple peaks, while the resonances for these same assignments in the apo and PPACK spectra were singlets. Often, one peak of the multiplet corresponded to the equivalent resonance in apo-thrombin (Supplementary Fig. 2). All of these assignments were verified by backbone walks using HNCA, HNCO, and HNCOCA spectra. The appearance of these multiplet resonances suggested that the presence of TM456 induces dynamics that are slow on the NMR timescale (< 80 s^-1^) in these residues.

### The TM binding site remains dynamic when TM is present

**Figure 1:**
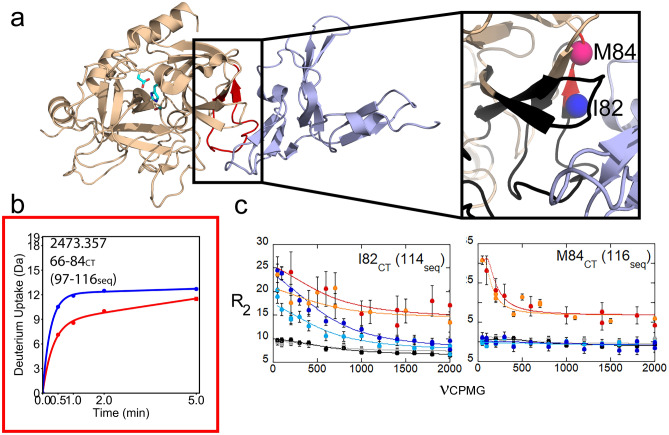
(**a**) Structure of thrombin (wheat) bound to TM456 (light blue) [PDB ID: 1DX5]. The side chains of the catalytic triad are shown as cyan sticks. Thrombin residues 66-84_CT_ (97-116seq) of the 70s_CT_ loop are colored red (left). A close-up of the 30s_CT_ and 70s_CT_ loops (right) has residues missing from the thrombin-TM456 HSQC colored black, and the amides in this strand for which CPMG data was obtained as spheres. For all figures, pink spheres indicate residues with NH resonances experiencing increased μs-ms dynamics in thrombin-TM456 and blue spheres indicate residues with NH resonances experiencing reduced μs-ms dynamics as compared to apo-thrombin. (**b**) Deuterium uptake plots for the peptide spanning residues 66-84_CT_ (97-116seq; MH + 2473.357) in apo-thrombin (blue) compared to thrombin bound to TM456 (red). Error bars (standard deviation of three replicates) are shown. (**c**) CPMG plots for resonances corresponding to Ile 82_CT_ (112seq) and Met 84_CT_ (116seq). For all figures, the red and orange curves are from spectra collected on thrombin-TM456 at 800 MHz and 600 MHz respectively, the blue and cyan curves are from apo-thrombin at 800 MHz and 600 MHz respectively, and the black and grey curves are from PPACK-thrombin at 800 MHz and 600 MHz respectively. For all figures, molecular structures were produced in PyMol, deuterium uptake plots were produced in DECAv112 ( available at https://github.com/komiveslab/DECA), NMR spectra were plotted in SPARKY (available at https://nmrfam.wisc.edu/nmrfam-sparky-distribution/), and NMR CPMG curves were plotted in Kaleidagraph v4.5.3.

Despite the stabilization of thrombin afforded by PPACK binding^20^, resonances corresponding to the N-terminus of the heavy chain Ile 16_CT_ (37seq) and nearly all of the residues in the 30s_CT_ (residues 54–61seq) and 70s_CT_ (residues 97–113seq) loops as well as residues 151-156_CT_ (193-197seq), and the 220s_CT_ loop 221-221A_CT_ (268-269seq) were not observed in previous NMR experiments on PPACK-thrombin or apo-thrombin^8,10^. Cross peaks for these residues were also not observed in the thrombin-TM456 spectra. We were, however, able to learn about the solvent accessibility/dynamics of these loops from HDX-MS, which reported on 99% of the thrombin sequence for experiments on WT-thrombin with TM456 either present or absent (Supplementary Fig. 3). The HDX-MS experiments showed a ~ 3 deuteron decrease in exchange of residues 66-84_CT_ (97-116seq) in the 70s_CT_ loop within 1 min when TM456 was present, consistent with the exclusion of solvent due to TM456 binding (Fig. 1a,b). Interestingly, this binding site “protection” was transient, becoming less over the 5 min time course of the HDX experiment suggesting that this region remains dynamic, which is consistent with our inability to observe resonances for the 70s_CT_ loop in the NMR. We did observe resonances for Ile 82_CT_ (114seq) and Met 84_CT_ (116seq) in the β-sheet following the 70s_CT_ loop and CPMG curves for these two residues showed that their chemical environments are changing on the μs-ms timescale (Fig. 1a,c).

### The thrombin N-terminal β-barrel becomes more dynamic when TM is bound

**Figure 2:**
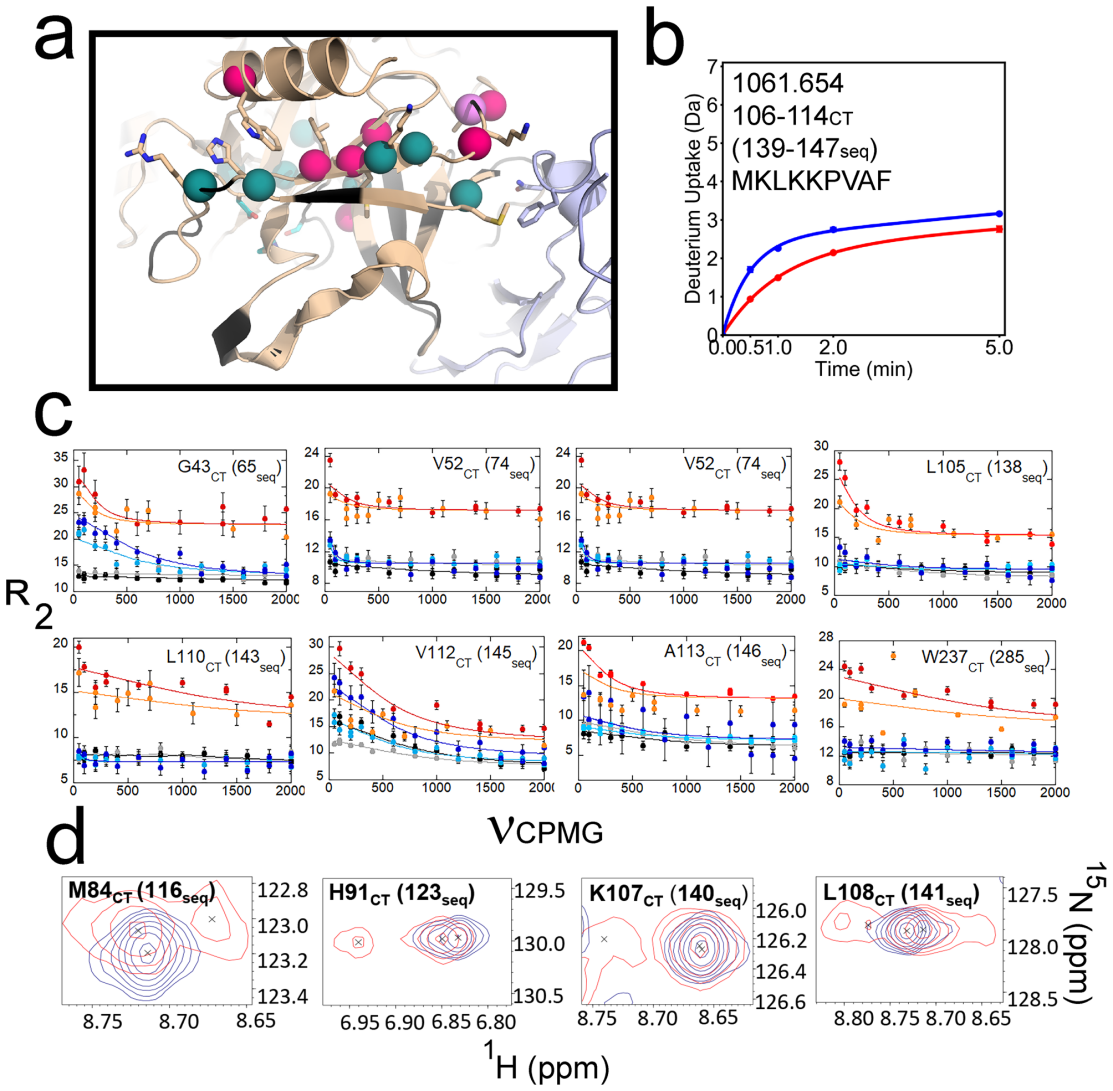
(**a**) Structure of thrombin (wheat) bound to TM456 (light blue) [PDB ID: 1DX5]. The residues corresponding to resonances missing from the thrombin-TM456 HSQC are colored black. The residues corresponding to multiplet resonances (teal spheres), resonances experiencing increased μs-ms dynamics in thrombin-TM456 compared to apo-thrombin (pink spheres), and resonances experiencing similar μs-ms dynamics in thrombin-TM456 and apo-thrombin (violet spheres) and the catalytic triad (cyan sticks) are shown. (**b**) Deuterium uptake plot for the peptide spanning residues 106-114_CT_ (139-147seq; MH + 1061.654). (**c**) CPMG plots for resonances in the thrombin N-terminal β-barrel (symbols as in Fig. 1). (**d**) Examples of thrombin-TM456 HSQC (red) doublet resonances compared to apo-thrombin (blue) resonances for residues in the N-terminal β-barrel.

Ten residues within the thrombin N-terminal β-barrel experienced more significant dynamics on the μs-ms timescale when TM456 was present. A subgroup of these residues was distributed along two antiparallel $\beta$-strands that connect the thrombin 70s_CT_ and 90s_CT_ loops (Fig. 2a). HDX-MS showed residues 106-114_CT_ (139-147seq) were more protected from deuterium exchange (by ~ 1 deuteron) when TM456 was present (Fig. 2b). Interestingly, CPMG experiments showed that TM456 induces μs-ms dynamics in four residues within this segment (Fig. 2c). This subgroup of residues also included Leu 105_CT_ (138seq), suggesting that the dynamic modulation induced by TM456 may be transferred into the catalytic Asp 102_CT_ (189seq) through these antiparallel β-strands. Resonances corresponding to Ile 88 and Tyr 89_CT_ (120-121seq) located directly across from Leu 105_CT_ and Met 106_CT_ in the neighboring β-strand could be assigned in the apo-thrombin spectrum, but they were absent in the thrombin-TM456 spectrum supporting the claim that the residues in this region are dynamic. Although we could assign Asp 102_CT_ (189seq), CPMG analysis could not be performed due to the presence of overlapped resonances. In addition, a few residues within these same antiparallel β-strands showed peak multiplets (Fig. 2d). Thus, TM456 appeared to stabilize the end of the β-strand leading to the catalytic Asp 102_CT_ (189seq), yet also induced μs-ms dynamics in this strand and the neighboring strand. These results provide strong evidence that TM binding at the 70s_CT_ loop induces dynamics along the antiparallel β-strands that connect the TM binding site to the active site catalytic residues.

### TM binding allosterically reduces the dynamics of the S1 pocket, and also induces a slow time scale conformational change in the C-terminal $\upbeta$-barrel

**Figure 3:**
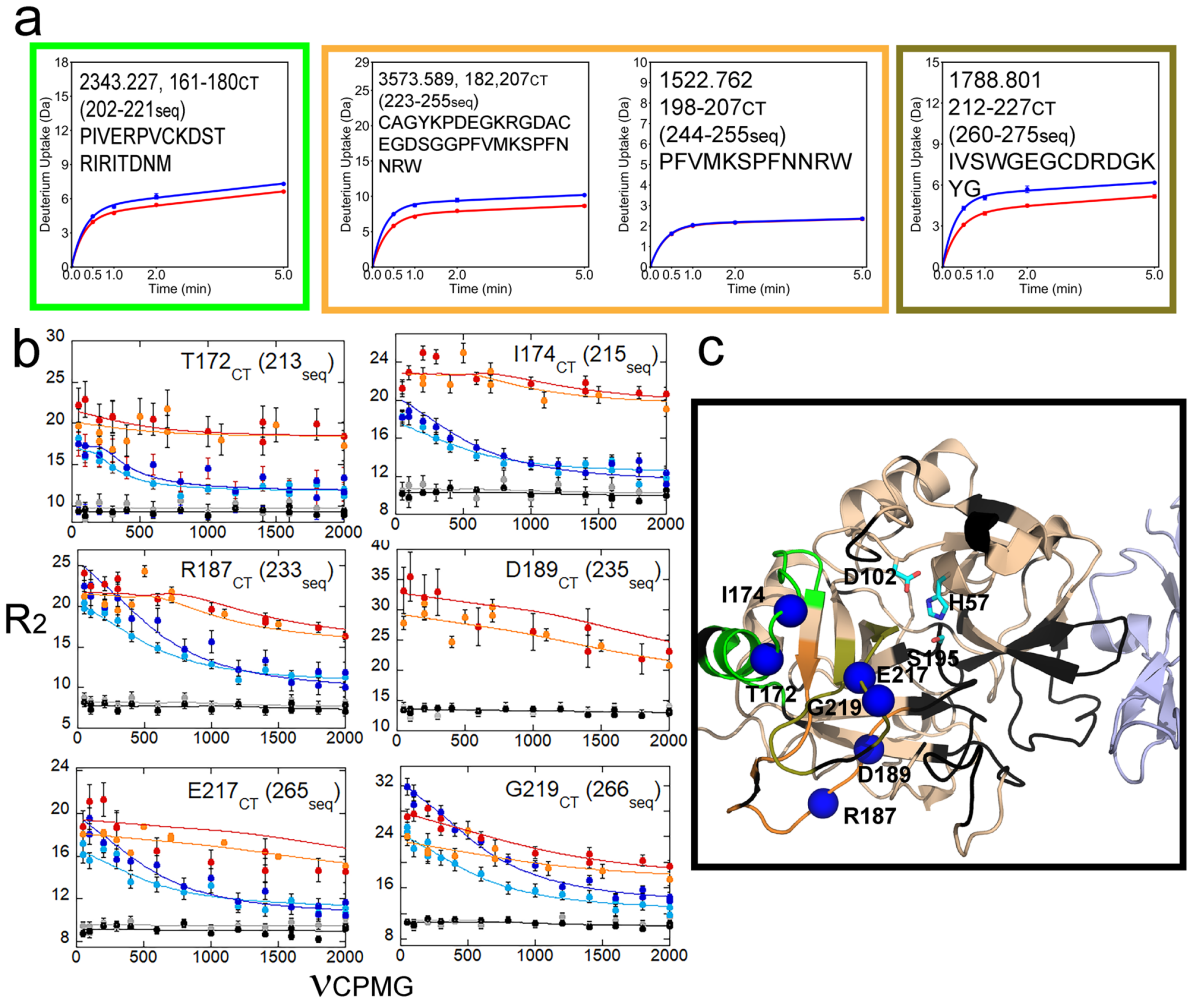
(**a**) Deuterium uptake plots for the peptides spanning residues 161-180_CT_ (202-221seq; MH + 2343.227), 182-207_CT_ (223-255seq; MH + 3575.589), 198-207_CT_ (244-255seq; MH + 1522.762), and 212-227_CT_ (260-275seq; MH + 1788.801). (**b**) CPMG plots for resonances experiencing reduced μs-ms dynamics around the S1 pocket when TM456 is bound compared to apo-thrombin (symbols as in Fig. 1). (**c**) Structure of thrombin (wheat) bound to TM456 (light blue) [PDB ID: 1DX5]. The residues within the peptides identified in panel (A) are colored accordingly. Residues with missing HSQC resonances (black) and residues corresponding to resonances experiencing reduced μs-ms dynamics in thrombin-TM456 compared to apo-thrombin (blue spheres) are shown.

HDX-MS revealed that TM456 binding reduced exchange in the 170s_CT_, 180s_CT_, and 220s_CT_ loops of thrombin despite the > 20 Å distance between the TM binding site and these loops. Upon TM456 binding, decreased exchange of ~ 1 deuteron was observed for the 170s_CT_ loop residues 161-180_CT_ (202-221seq) and of ~ 2 deuterons for the 180s_CT_ loop residues 182-198_CT_ based on subtraction of deuterium uptake into residues 198-207_CT_ (244-255seq) from uptake into residues 182-207_CT_ (223-255seq). Finally, a decreased exchange of ~ 1.5 deuterons was observed for the 220s_CT_ loop residues 212-227_CT_ (260-275seq) (Fig. 3a,c). CPMG analysis identified seven residues within these loops that showed significantly decreased μs-ms dynamics when TM456 was present as compared to apo-thrombin (Fig. 3b,c).

**Figure 4:**
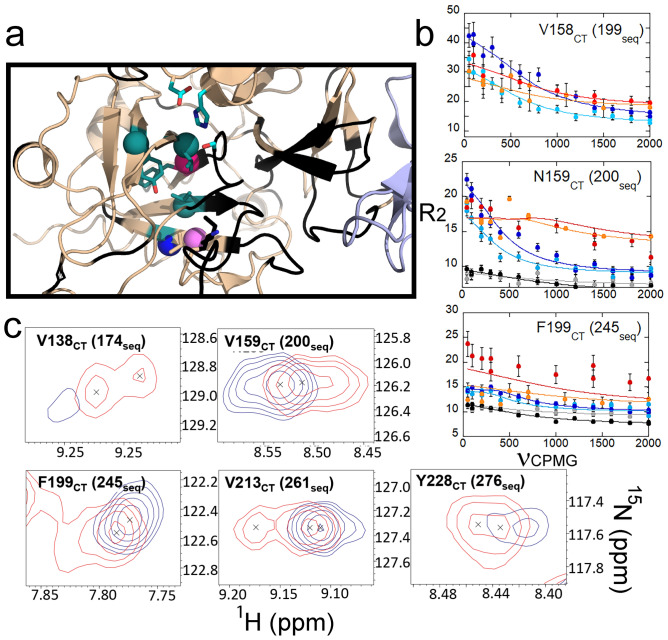
(**a**) Structure of thrombin (wheat) bound to TM456 (light blue) [PDB ID: 1DX5] showing the C-terminal β-barrel. Residues with missing resonances (black), doublet resonances (teal spheres), resonances experiencing increased μs-ms dynamics (pink spheres), resonances experiencing similar μs-ms dynamics (violet spheres) and resonances experiencing decreased μs-ms dynamics (blue spheres) in thrombin-TM456 compared to apo-thrombin are shown. (**b**) CPMG plots for resonances in the thrombin C-terminal β-barrel (symbols as in Fig. 1). (**c**) Examples of thrombin-TM456 HSQC (red) doublet resonances compared to apo-thrombin (blue) resonances.

Val 158_CT_ and Asn 159_CT_ are in the center of the β-strand following the 140s_CT_ loop and leading to the 170s_CT_ loop (Fig. 4a). A stack of three valines (residues 158_CT_ (199seq), 138_CT_ (174seq) and 213_CT_ (261seq)) forms one side of the hydrophobic core of the C-terminal β-barrel (Fig. 4a). Val 158_CT_ (199seq) experiences different chemical environments on the μs-ms timescale in all three thrombin states (Fig. 4b). Val 138_CT_ (174seq) also showed μs-ms timescale dynamics in apo thrombin, but this resonance became a doublet in the thrombin-TM456 HSQC spectrum (Fig. 4c). Finally, V213_CT_ (261seq) was also a doublet only in the thrombin-TM456 HSQC spectrum (Fig. 4c). Adjacent to Val 158_CT_, Asn 159_CT_ was a doublet only in the thrombin-TM456 complex, and it was experiencing different chemical environments on the μs-ms timescale in apo-thrombin but not in the thrombin-TM456 complex (Fig. 4b,c). The core of the C-terminal β-barrel is flanked by Phe 199_CT_ (245seq) which also was a doublet in the thrombin-TM456 HSQC spectrum and showed μs-ms timescale dynamics in all three thrombin states (Fig. 4b,c). Tyr 228_CT_ (276seq) could be assigned to both a doublet resonance and a singlet resonance with notably different chemical shifts (Supplementary Fig. 4). Thus, the addition of TM456 causes decreased H/D exchange in the loops around the thrombin S1 pocket, reduces μs-ms timescale dynamics, and also induces dynamics that are slow on the NMR timescale in the residues that make up the hydrophobic core of the C-terminal β-barrel.

### TM induces slow dynamics in the N-terminus of the heavy chain and the 140s_CT_ loop

**Figure 5:**
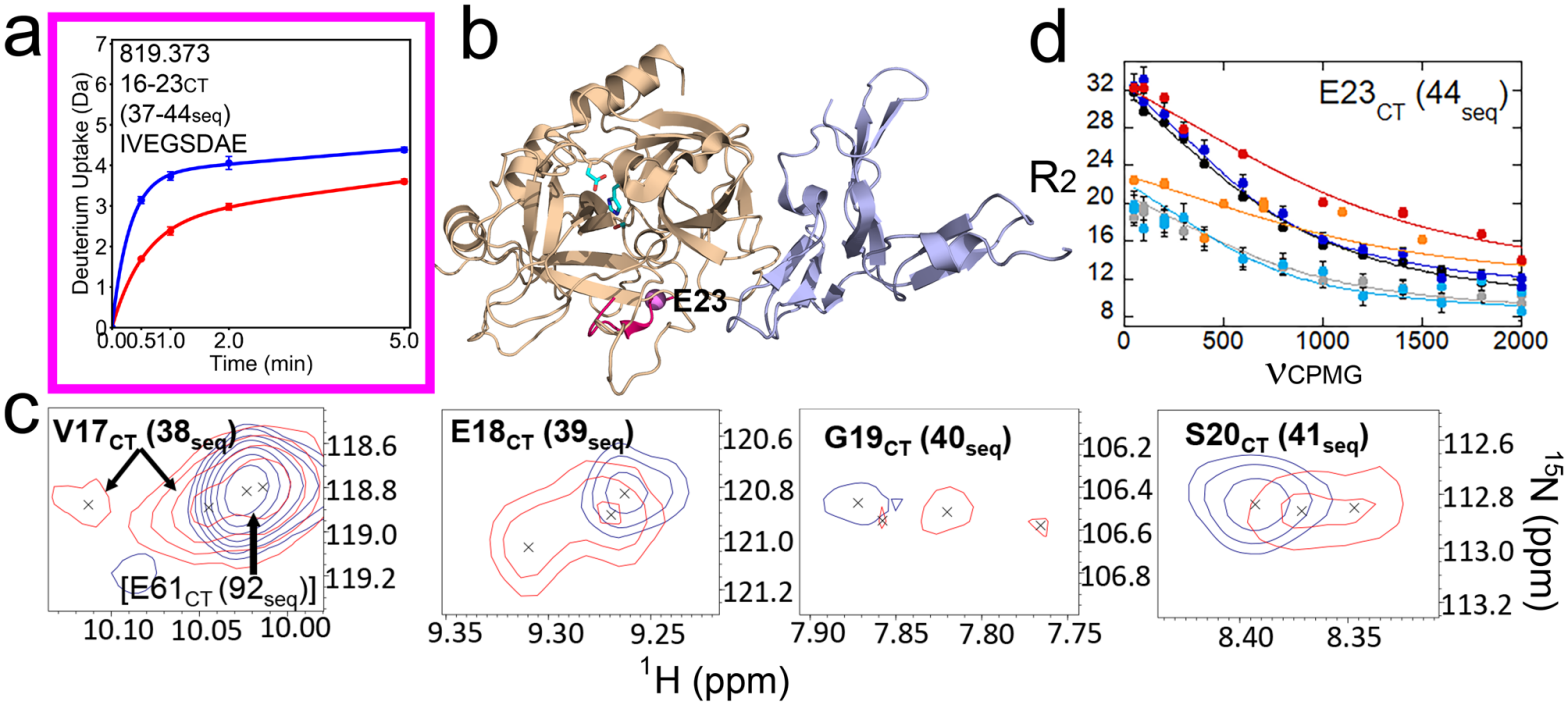
(**a**) Deuterium uptake plot for the peptide spanning residues 16-23_CT_ (37-44seq; MH + 819.373). (**b**) Location of residues 16-23_CT_(37-44_seq_) in the structure of thrombin (wheat) bound to TM456 (light blue) [PDB ID: 1DX5]. (hot pink). The resonance corresponding to Glu 23_CT_ (44seq) experienced similar μs-ms dynamics in thrombin-TM456 and apo-thrombin (purple sphere). (**c**) Examples of thrombin-TM456 HSQC (red) doublet resonances compared to apo-thrombin (blue) resonances for residues at the N-terminus of the heavy chain. (**d**) CPMG plot for the resonance corresponding to residue Glu 23_CT_ (44seq) (symbols as in Fig. 1).

HDX-MS showed that residues 16-23_CT_ (36-44seq) at the N-terminus of the thrombin heavy chain showed decreased exchange when TM456 was bound to thrombin (Fig. 5a). The x-ray crystal structure of thrombin-TM456 (PDB ID: 1DX5^17^), shows the hydrophobic side chains of Val 158_CT_ (199seq) and Val 138_CT_ (174seq) interacting with the side chain of Ile 16_CT_ (36seq) at the N-terminus of the thrombin heavy chain, which inserts into the Ile cleft to form the catalytically active conformation. Adjacent to the new N-terminus belonging to Ile 16_CT_, cross peaks for Val 17_CT_- Ser 20_CT_ (38-41seq) also were doublets in the thrombin-TM456- spectrum (Fig. 5b). The cross peak for Val 17_CT_ (38seq) could not be assigned in apo-thrombin, but it could be assigned in the thrombin-TM456 spectrum suggesting that it adopts a more defined conformation(s) in the thrombin-TM456 complex. Glu 23_CT_ (44seq) showed dynamics on the μs-ms timescale in all three thrombin states, providing more evidence that the N-terminus of the heavy chain remains somewhat mobile in the thrombin-TM456 complex (Fig. 5c). Thus, TM456 modulates the dynamics of the N-terminus of the heavy chain decreasing amide exchange and inducing dynamics that are slow on the NMR time scale.

**Figure 6:**
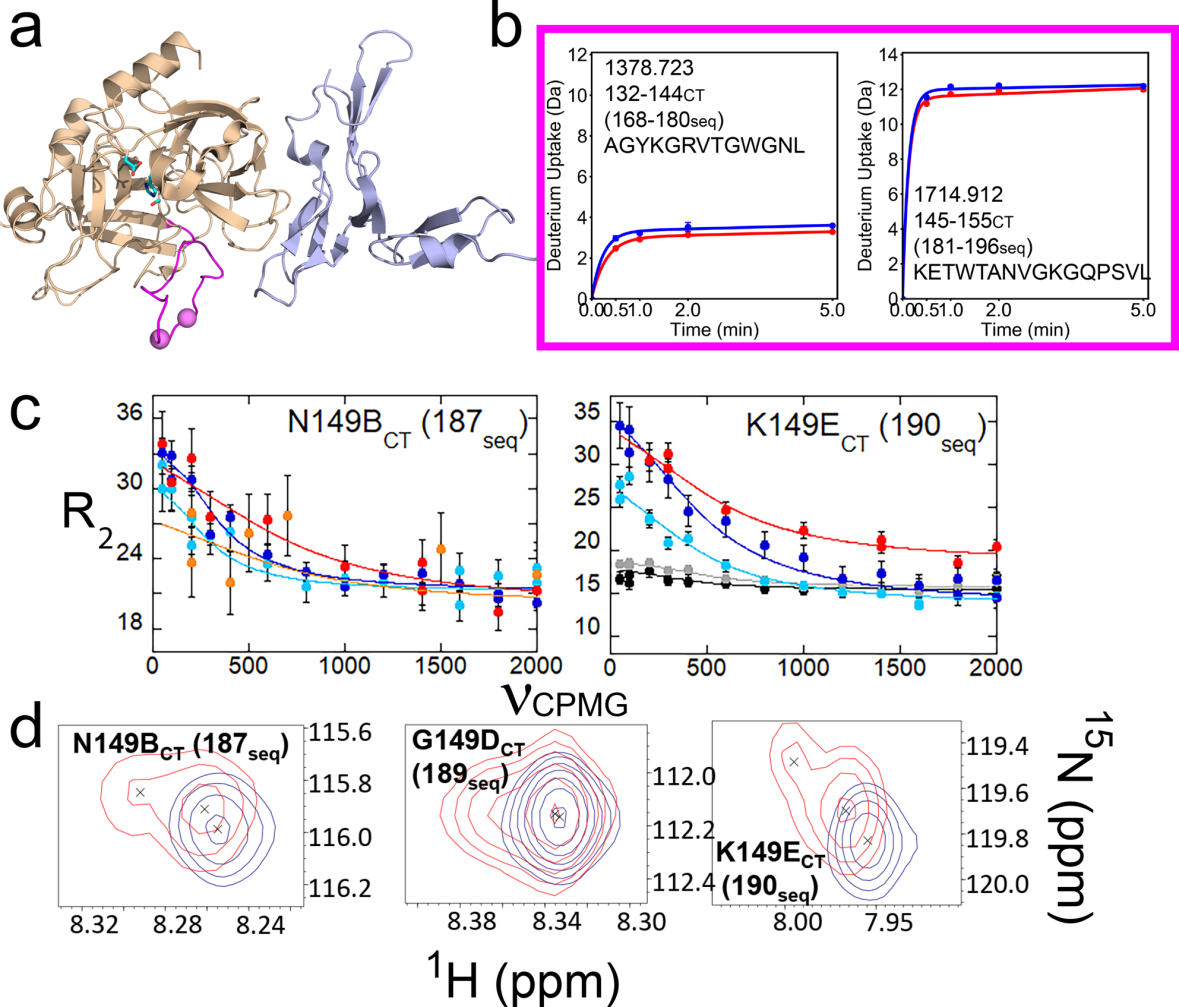
(**a**) Structure of thrombin (wheat) bound to TM456 (light blue) [PDB ID: 1DX5]. Residues 140-155_CT_ (176-196seq) of the 140s_CT_ loop are colored magenta. The amides of residues corresponding to resonances experiencing μs-ms dynamics in both thrombin-TM456 and apo-thrombin are shown as violet spheres. Sidechains are shown for the catalytic triad (cyan). (**b**) Deuterium uptake plots for the peptides spanning residues 132-144_CT_ (168-180seq; MH + 1378.723) and 145-155_CT_ (181-196seq; MH + 1714.912). The colors are the same as in previous figures. (**c**) CPMG plots for thrombin resonances corresponding to residues Asn 149B_CT_ (187seq) and Lys 149E_CT_ (190seq). The colors of the curves are the same as in previous figures. CPMG data could not be obtained from the 600 MHz spectra for Thr 149_CT_ (187seq). (**d**) Examples of thrombin-TM456 HSQC (red) doublet resonances compared to apo-thrombin (blue) resonances for residues in the 140s_CT_ loop.

Residues 139-149A_CT_ (175-186seq), the N-terminal half of the 140s_CT_ loop could not be assigned in any thrombin samples (Fig. 6a). The dynamics of this part of the 140s_CT_ loop were determined instead from HDX-MS experiments. Residues 145-155_CT_ (181-196seq) showed nearly complete exchange, consistent with this loop being very dynamic and solvent-exposed whereas residues 132-144_CT_ (168-180seq) exchanged much less (Fig. 6b). The deuterium exchange of the 140s_CT_ loop did not change with the addition of TM456. Residues N149B_CT_ and K149E_CT_ at the tip of the 140s_CT_ loop could be assigned and CPMG data revealed they were undergoing similar μs-ms timescale dynamics in all thrombin states (Fig. 6c). Residues 149B-150_CT_ (187-191seq) all became doublets in the thrombin-TM456 HSQC (Fig. 6d) but were singlets in the other thrombin forms. Thus, TM456 induces slow timescale dynamics in the C-terminal half of the 140s_CT_ loop.

## Discussion

For thrombin to catalyze fibrinogen cleavage, fibrinogen must engage both the anion binding exosite 1 (where TM binds) and the active site^25^. It is thought that TM promotes cleavage of protein C by engaging anion binding exosite 1 in a manner similar to fibrinogen. Thus, one can think of the thrombin-TM complex as revealing the catalytically active form of thrombin via engagement of anion binding exosite 1. Previous studies have highlighted the dynamics of thrombin loops^7,9,11, 26^, and suggested that loop dynamics may allow it to bind different substrates. This has led some to speculate that TM may take advantage of modulating loop dynamics to direct catalysis toward protein C, its anticoagulant substrate^26–29^. Here we present a combination of HDX-MS and NMR CPMG data yielding a much more complete understanding of dynamics throughout the thrombin-TM complex. The rich dynamic behavior of the thrombin-TM complex appears to be related to the complicated serine protease multi-step catalytic mechanism.

A remarkable finding is that entropy compensation appears to occur between the N-terminal and C-terminal β-barrels of thrombin when TM binds. Ten residues showed increased dynamics in the N-terminal β-barrel when compared to apo- and PPACK thrombin. Conversely, nine residues showed decreased dynamics in the C-terminal β-barrel. Thus, TM appears to order the C-terminal β-barrel and disorder the N-terminal β-barrel to which it is bound. This result was completely unexpected considering that TM binding reduces amide exchange throughout thrombin.

HDX-MS experiments showed reduced exchange at the TM binding site, but this could be due either to reduced dynamics or to interface protection^6^. The H/D exchange protection did not persist after 5 min of incubation, which is likely due to the very rapid association and dissociation kinetics observed for thrombin-TM binding^18^. NMR signals were not observed for much of the 70s_CT_ loop in apo and PPACK-thrombin^8^, and here we show that these cross peaks are also missing even when TM is bound to thrombin. The thrombin-TM interaction is entirely entropically-driven^20^, which is also consistent with the binding site remaining disordered. Apo thrombin has a large number of residues that experience different chemical environments on the μs-ms timescale throughout the molecule^8^.

**Figure 7:**
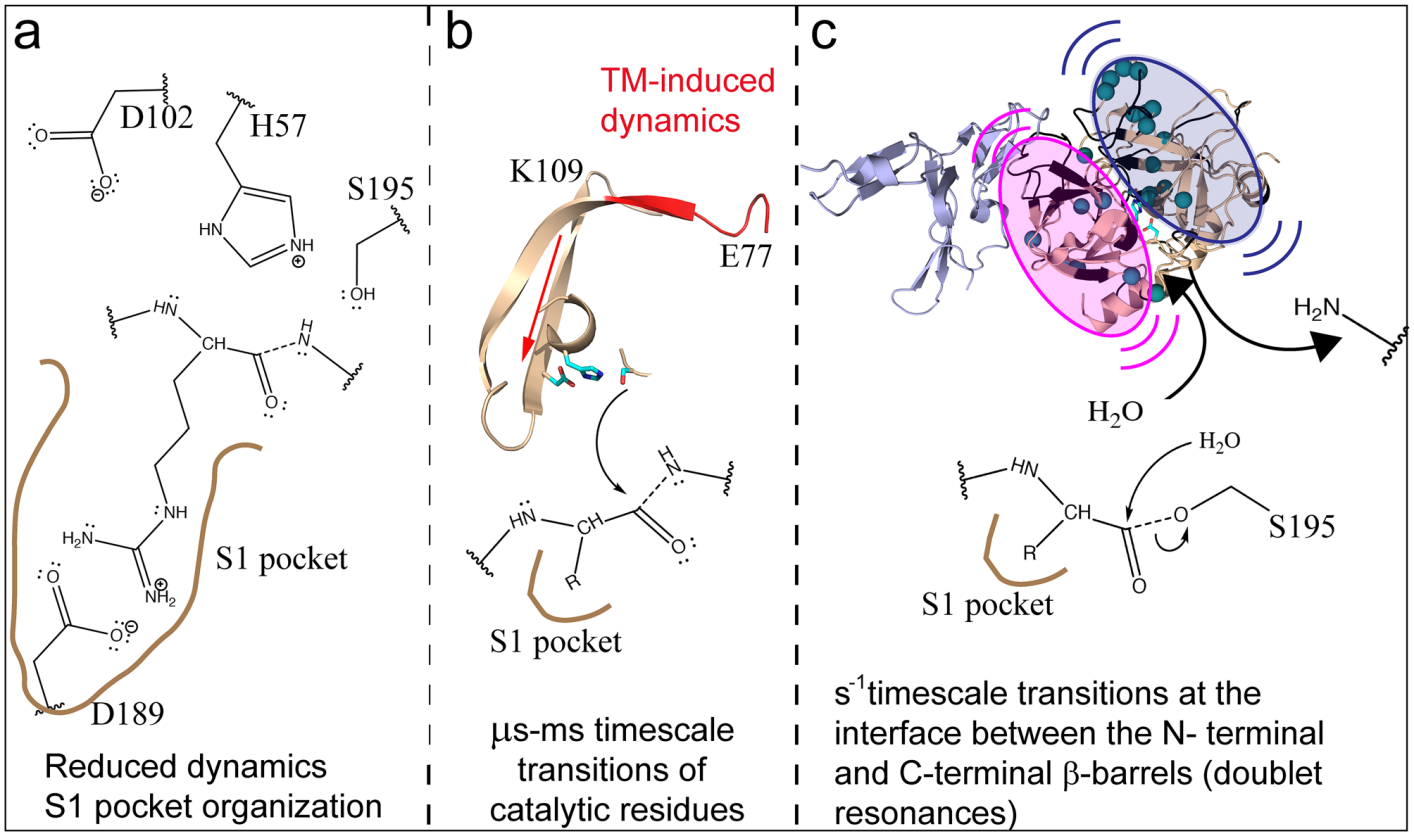
A model that describes the steps of the serine protease mechanism. (**a**) TM decreases dynamics in the substrate binding pocket to promote protein C binding. (**b**) TM-induces μs-ms dynamics that may promote the optimal activity of the catalytic triad. The backbone of residues 77-84_CT_ (108-116seq) is colored red, and the backbone of residues 56-58_CT_ (78-80seq), 85-113_CT_ (117–146), and 195_CT_ (241seq) are colored wheat. The side chains of the catalytic triad are shown as cyan sticks. (**c**) Doublet resonances indicate TM induced dynamics on the seconds time scale of the N-terminal (pink oval) and C-terminal (blue oval) β-barrels that may be required for the release of the first product from Ser 195_CT_ and entry of H_2_O into the active site for acyl enzyme hydrolysis.

Interestingly, the reduction in μs-ms timescale dynamics observed in the C-terminal β-barrel involves residues in the loops implicated in the formation of the S1 pocket^12,13^. Six residues within these loops were experiencing different chemical environments on the μs-ms timescale in apo-thrombin but their dynamics were quenched by binding PPACK. Remarkably, the dynamics of all of these residues were also quenched in the thrombin-TM complex, strongly indicating that TM binding over 25 Å away allosterically organizes the S1 pocket. These residues include Asp 189 at the bottom of the S1 substrate binding pocket and the sodium-binding site. The fact that this part of thrombin is significantly rigidified by TM was also observed through crystallographic studies on thrombin-TM in the absence of Na^+^^29^, and explains the insensitivity of the thrombin-TM complex to Na^+^ concentration ^30^, as well as the increased binding rate of thrombin active-site inhibitors observed when TM is bound ^31–35^. Such a pre-formation of this critical substrate-binding pocket may account for the marked improvement of the association rate of protein C into the thrombin active site from 27 M^-1^ s^-1^ in the absence of TM to 10^5^ M^-1^ s^-1^ in the presence of TM (Fig. 7a)^36^.

The next step in the proteolytic cleavage of protein C by thrombin is formation of the first covalently-bound intermediate. This intermediate is probably mimicked by the PPACK-thrombin complex^6–9,11^, which was observed to have the lowest degree of conformational heterogeneity^7,8^. A similar conformationally rigid, covalently-bound intermediate species was recently reported for the UMP/CMP kinase from *D. discoideum*^37^, suggesting that rigidity may be a common feature of covalently-bound reaction intermediates.

TM improves the *k*_cat_ for thrombin cleavage of protein C by over 200 fold^38^. We found that TM induces dynamics in a pair of β-strands that link the TM binding site to the catalytic residues through the N-terminal β-barrel. This pair of β-strands, which connect the 70s_CT_ and 90s_CT_ loops, was only dynamic in the thrombin-TM complex, but not in apo-thrombin or PPACK-thrombin. Community network analysis of accelerated MD simulations had indicated that several small communities of residues were moving independently of one another in this region of apo-thrombin, whereas these communities coalesce into a single community when TM binds^11^. We now have evidence that these predicted correlated motions involve the pair of β-strands, one of which leads to the catalytic Asp 102_CT_ (189seq). It is interesting to speculate that these motions may be important for catalysis since Asp 102_CT_ must closely approach His 57_CT_ in order to raise its pKa to promote formation of both tetrahedral intermediates in the catalytic mechanism (Fig. 7b).

Remarkably, many cross peaks in the HSQC were doublets only in thrombin-TM456 but not in apo-thrombin or PPACK thrombin. When we mapped all of the residues for which doublets are observed onto the structure of thrombin, we saw that they fall all along the interface between the N-terminal and C-terminal β-barrels (Fig. 7c). Doublets represent dynamics that are slow on the NMR time scale, < 80 s^-1^ yet this is still fairly fast compared to the *k*_cat_ for protein C cleavage of 5 s^-1^^16^. It is tempting to speculate that the doublets reveal thrombin dynamics that are important for catalysis of the slow steps of peptide bond cleavage. For serine proteases, the slow steps are thought to be release of the first product, the N-terminal portion after cleavage of the scissile bond, and subsequent binding of a water molecule that is required for hydrolysis of the acyl-enzyme intermediate which remains bound in the S1 pocket (Fig. 7c).

These data support a model of the serine protease reaction coordinate that explains how the conformational heterogeneity induced by TM facilitates effective catalysis of protein C activation. TM binding allosterically remodels apo-thrombin dynamics to accomplish three catalytic functions: (1) TM decreases dynamics in the C-terminal β-barrel forming the S1 pocket to accelerate protein C binding, (2) TM enhances μs-ms timescale dynamics in a pair of β-strands connecting the TM binding site to the 90s_CT_ loop in the N-terminal β-barrel. The dynamics of these strands likely alter Asp 102_CT_ to promote catalysis, and (3) TM causes dynamics that are slow on the NMR timescale along the interface between the N-terminal and C-terminal β-barrels. These slower time scale dynamics are likely required for release of the first product and subsequent hydrolysis of the covalently bound acyl-enzyme intermediate.

## Methods

### Thrombin expression and purification

 The S195M mutant and WT human thrombin were expressed and refolded from *E. coli* as previously described^8,10^. After initial refolding and initial purification, the S195M thrombin was mixed with WT thrombin at a ratio of 1:30 WT:S195M and was left to rock for 12–16 h at 30 °C to convert the meizothrombin species formed to α-thrombin. After activation, the WT α-thrombin was removed by addition of biotinyl-PPACK (Haematologic Technologies) then captured on streptavidin resin (Thermo Scientific) and removed. The α-form of the S195M thrombin was isolated from other forms by chromatography on a 10/100 GL MonoS cation exchange column (GE Healthcare Life Sciences) using a gradient of 100 mM–500 mM NaCl in 25 mM phosphate pH 6.5. This method of thrombin purification has been shown to result in > 95% α-thrombin, and previous NMR analysis of isotopically labeled thrombin prepared this way demonstrated that the species present was α-thrombin^10,18^.

### Production of TM456

 The TM456 species used for all thrombin-TM456 experiments was “TM456m” which ends at Gly449 and has Cys448 changed to Ser as previously described^16^. Protein C activation assays were performed to assess the specific activity of the expressed TM456 as previously described^16^.

### Hydrogen–deuterium exchange mass spectrometry

 The α-thrombin-TM456 complex was prepared at a molar ratio of 1:10 thrombin:TM456, to ensure 99% of thrombin would be bound to TM456 at the protein concentrations used in the experiment. HDX-MS was performed using a Waters Synapt G2Si system with HDX technology (Waters Corporation)^39^. Deuterium exchange reactions were measured in triplicate and experiments were performed and analyzed as described previously^40^. HDX-MS was performed using a Waters Synapt G2Si system with HDX technology (Waters Corporation)^39^. Deuterium exchange reaction were prepared using a Leap HDX PAL autosampler (Leap technologies, Carrboro, NC). D_2_O buffer was prepared by lyophilizing 1 mL of 250 mM phosphate pH 6.5 along with 850 mM NaCl for the apo-thrombin experiments and 1000 mM NaCl for the thrombin-TM456 experiments, before being resuspended in 10 mL 99.96% D_2_O immediately before use. For each deuteration time point (performed in triplicate), 5 µL of protein was held at 25 °C for 5 min before being mixed with 55 µL of D_2_O buffer, then quenched for 1 min at 1 °C by mixing 1:1 with ice cold 250 mM TCEP pH 2.5. The quenched sample was then injected into a 50 µL sample loop, followed by digestion on an in-line pepsin column (immobilized pepsin, Pierce, Inc.) at 15 °C. The resulting peptides were captured on a BEH C18 Vanguard pre-column, separated by analytical chromatography (Acquity UPLC BEH C18, 1.7 µM, 1.0 × 50 mm, Waters Corporation) using a 7–50% acetonitrile in 0.1% formic acid over 7.5 min, and electrosprayed into the Waters Synapt G2Si quadrupole time-of-flight mass spectrometer. The mass spectrometer was set to collect data in the Mobility, ESI^+^ mode; mass acquisition range 200–2,000 (m/z); scan time 0.4 s with continuous lock mass correction. For peptide identification, the mass spectrometer was set to collect data in MS^E^, mobility ESI + mode and 10 μM α-thrombin. Peptides masses were identified using PLGS 2.5 (Waters Corporation) requiring a minimum number of 250 ion counts for low energy peptides and 50 ion counts for their fragment ions; the peptides also had to be larger than 1500 Da.

DynamX 3.0 (Waters Corporation) was used to determine deuterium uptake based on the peptides identified by PLGS, but incorporating additional filters including a cut-off score of 7, minimum products per amino acid of 0.2, maximum MH + error tolerance of 5 ppm, retention time standard deviation of 5%, and requiring that the peptide be present in at least 2 of the 3 peptide identification runs. The deuterium uptake for each peptide was calculated by comparing the centroids of the mass envelopes of the deuterated samples vs. the undeuterated controls. For all HDX-MS data, at least 2 biological replicates were analyzed, each with 3 technical replicates. Data are represented as mean values + /- SEM of 3 technical replicates due to processing software limitations, however biological replicates were highly reproducible due to use of the LEAP robot for all experiments. The deuterium uptake was corrected for back-exchange using a global back exchange correction factor (typically 25%) determined from the average percent exchange measured in disordered termini of various proteins. ANOVA analyses and t tests with a p value cutoff of 0.05 implemented in the program, DECA, were used to determine the significance of differences between HDX data points^40^. The peptides reported on the coverage maps are actually those from which deuterium uptake data were obtained. Deuterium uptake plots were generated in DECA (github.com/komiveslab/DECA).

### NMR sample preparation

 Purified ^2^H-^15^ N-S195M or ^2^H-^15^ N-^13^C-S195M thrombin was added to unlabeled TM456 at a molar ratio of 1:1.5 thrombin:TM456 to ensure 99% of thrombin would be bound to TM456 at the protein concentrations used in the experiment. The proteins were suspended in buffer containing 25 mM phosphate pH 6.5,150 mM NaCl, 0.05% sodium azide and 10% D_2_O at a final concentration of 0.12 mM thrombin.

### NMR data collection

 For all experiments, Shigemi salt-tolerant susceptibility matched NMR tubes were used. The sample volume was 170 μL and the tube was properly oriented inside the magnet to optimize S/N and minimize sample heating ^41^. All NMR experiments were acquired on Varian VNMRS (Agilent Technologies, Santa Clara, CA) and Bruker Avance III spectrometers operating at 600, 800 and 900 MHz (1H), and equipped with cryogenic triple-resonance probes at 298 K.

For the sequence specific assignment of backbone resonances, the TROSY version of 2D ^1^H,^15^ N-HSQC and 3D HNCA, 3D HN(CO)CA and 3D HNCO spectra were recorded^42,43^. The acquisition parameters for these spectra are reported in Table S1. The 3D spectra were recorded with Non-Uniform Sampling (NUS) using optimized Poisson-gap distribution schedules^44^ with sampling rates ranging between 36 and 38%. Spectra were processed using NMRPipe. The 3D spectra recorded with NUS were reconstructed using the SMILE package available in NMRPipe^45^, and analyzed with NMRFAM-SPARKY.

To identify conformational exchange, spectra were recorded at 600 and 800 MHz (^1^H) using a CPMG relaxation dispersion experiment with TROSY selection. The total relaxation delay for CPMG was fixed to 40 ms and multiple 2D spectra were recorded in an interleaved manner by changing the number of CPMG pulses within the constant relaxation delay. A total of 10 ʋ_CPMG_ values ranging from 50 to 2000 Hz were recorded both at 600 and 800 MHz, with 2 additional ʋ_CPMG_ values recorded in duplicate for error estimation. All relaxation dispersion spectra were processed using NMRPipe and peak intensities were extracted using NMRFAM-SPARKY^46^.

### NMR resonance assignments

 Assignments were transferred from the previously assigned PPACK-thrombin^10^ and apo-thrombin^8^. All transferred and new assignments were confirmed with the 3D experimental data. Twenty-seven residues for which N–H peaks were visible in PPACK-bound thrombin did not have visible peaks in the HSQC-TROSY spectrum of thombin-TM456. These included residues: Gln 30_CT_ (51_seq_), Val 66_CT_ (97_seq_), Lys 81_CT_ (113_seq_), Arg 97A – Leu 99_CT_ (130-132_seq_), Thr 139 – Ala 149A_CT_ (175-186_seq_), Gln 151 – Gln 156_CT_ (192-197_seq_), Glu 192_CT_ (238_seq_), Gly 196_CT_ (242_seq_), Asp 221_CT_ (268_seq_), and Arg 221A_CT_ (269 _seq_). Four resonances were visible in the HSQC-TROSY spectrum of apo-thrombin, but these were not observed in the thrombin-TM456m spectrum. These included resonances for residues: Ser 45_CT_ (67_seq_), Val 66_CT_ (97_seq_), Ile 88_CT_ (120_seq_), and Tyr 89_CT_ (121_seq_). Nine residues were visible in the HSQC-TROSY thrombin-TM456m spectrum that were not observed in the apo-thrombin spectrum. These included residues: Glu 18_CT_ (38_seq_), Gly 188 – Cys 191_CT_ (234-237_seq_), Trp 215_CT_ (263_seq_), Cys 220_CT_ (267_seq_), Tyr 225_CT_ (273_seq_), and Gly 226_CT_ (274_seq_).

### CPMG Experiments

The effective relative relaxation rates (*R*_*2,eff*_) due to contributions from conformational exchange on μs-ms time scale were evaluated with CPMG experiments collected at NMRFAM using a TROSY-CPMG pulse sequence^47^. The 600 MHz and 800 MHz *R*_*2,eff*_ relaxation dispersion data for all thrombin-TM456 residues with *R*_ex_ > 6 Hz (24 residues) were individually fit to the Richard-Carver Eq. ^48^ by using the software package CPMGFit (http://www.palmer.hs.columbia.edu/software/cpmgfit.html). We attempted to globally fit the thrombin-TM456 CPMG data using GLOVE, which minimizes global and local parameters alternately, and incorporates a Monte-Carlo minimization method to allow fitting parameters to pass through local minima^49^. However, none of the residues with *R*_ex_ > 6 s^−1^ could be globally fit within an acceptable χ^2^ value.

## Supplementary Information

Supplementary Information

## Data Availability

The Chemical Shift Assignments for thrombin in complex with TM456 have been deposited with BMRB ID 50,678.
