# Supplementary material for: Serine protease dynamics revealed by NMR analysis of the thrombin-thrombomodulin complex
Source: Sci Rep. 2021 Apr 30;11:9354. doi: 10.1038/s41598-021-88432-z (PMC8087772; doi:10.1038/s41598-021-88432-z)
Supplement: Supplementary file 1 — Supplementary Information [file 41598_2021_88432_MOESM1_ESM.pdf]

Supplementary Information for

Serine protease dynamics revealed by NMR analysis of the thrombin-thrombomodulin complex

Riley B. Peacock[1], Taylor McGrann[1] Marco Tonelli[2], and Elizabeth A. Komives[1]*

[1]Department of Chemistry and Biochemistry, University of California, San Diego, 9500 Gilman Drive, La Jolla, CA 92093-0378.

[2] NMRFAM University of Wisconsin, 433 Babcock Drive, Madison, WI 53706.

*Corresponding author: Elizabeth A. Komives

Department of Chemistry and Biochemistry, University of California, San Diego, 9500 Gilman Drive, La Jolla, CA 92092-0378*Paste corresponding author name(s) here.

Email: ekomives@ucsd.edu

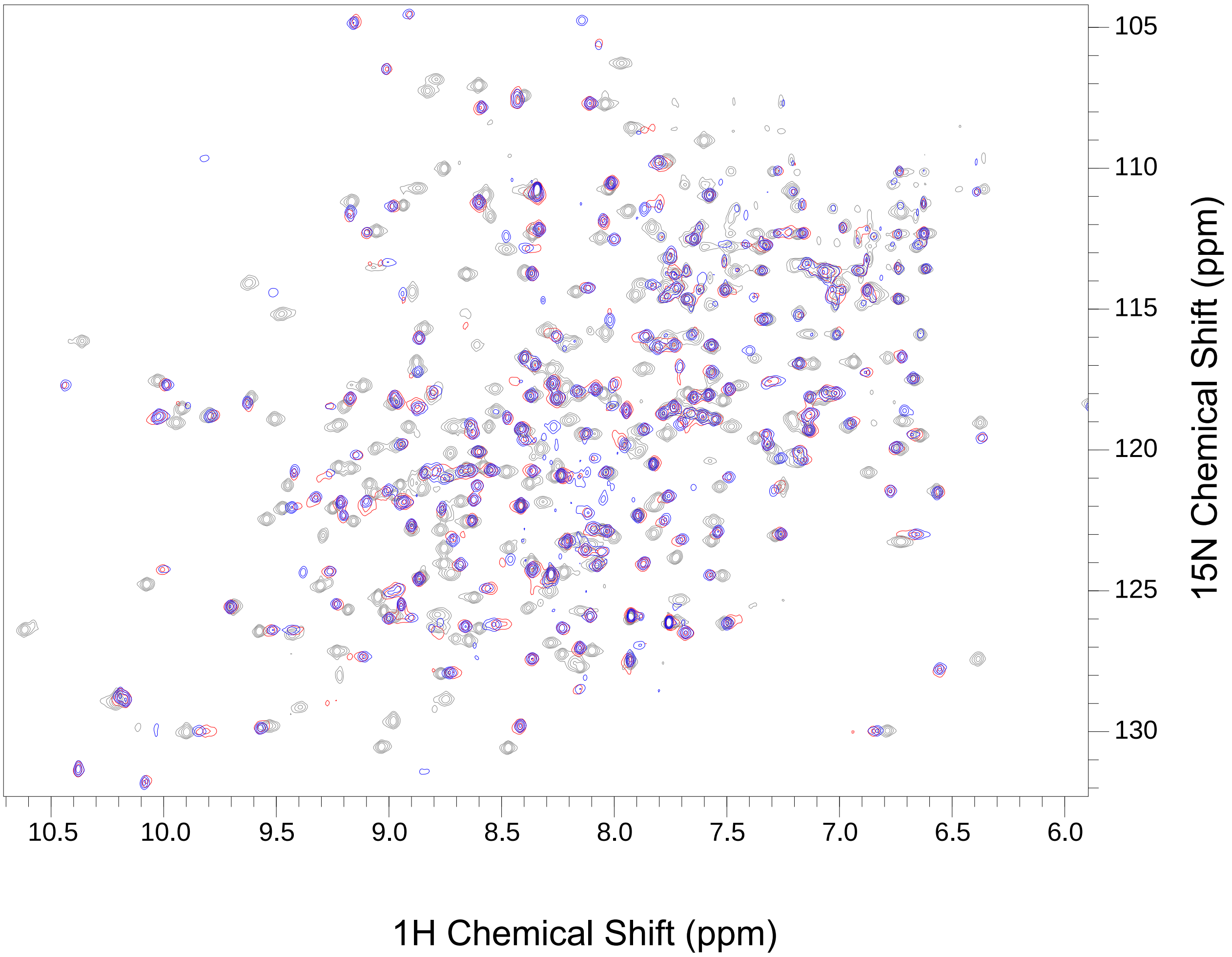

Fig. S1. TROSY HSQC Spectra of apo thrombin (blue), thrombin inhibited with D-Phe-Pro-Arg chloromethylketone (PPACK) (grey) and the thrombin-TM456 complex (red). All spectra were acquired at 800 MHz, room temperature, and a concentration of 0.12 mM.

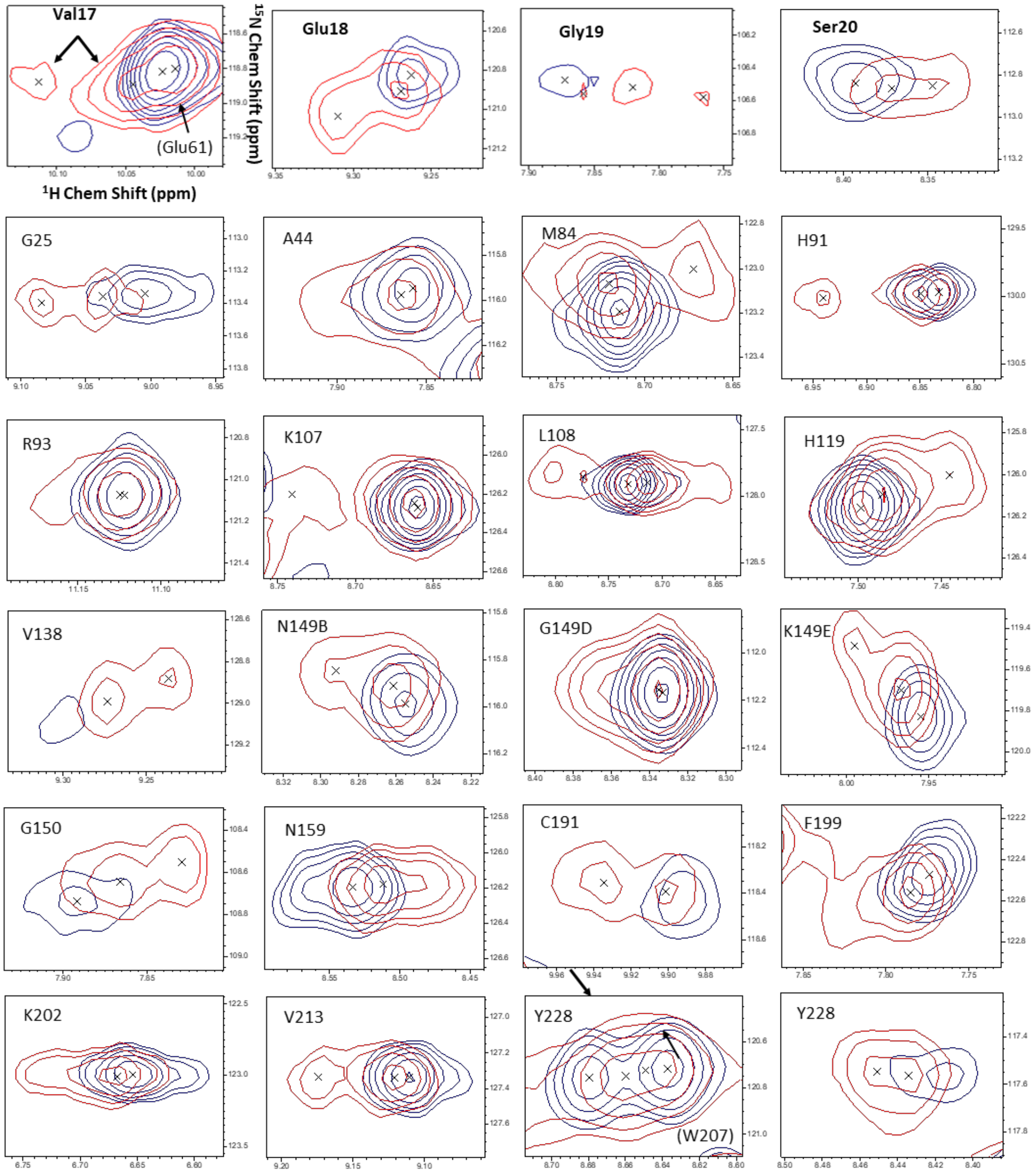

Fig. S2. All thrombin resonances that appeared as doublets or multiplets in the thrombin-TM456 HSQC spectrum. Residues are numbered according the the CT numbering scheme. The red traces correspond to the thrombin-TM456 HSQC spectrum taken at 800 MHz, while the blue traces correspond to the apo-thrombin spectrum taken at 800 MHz.

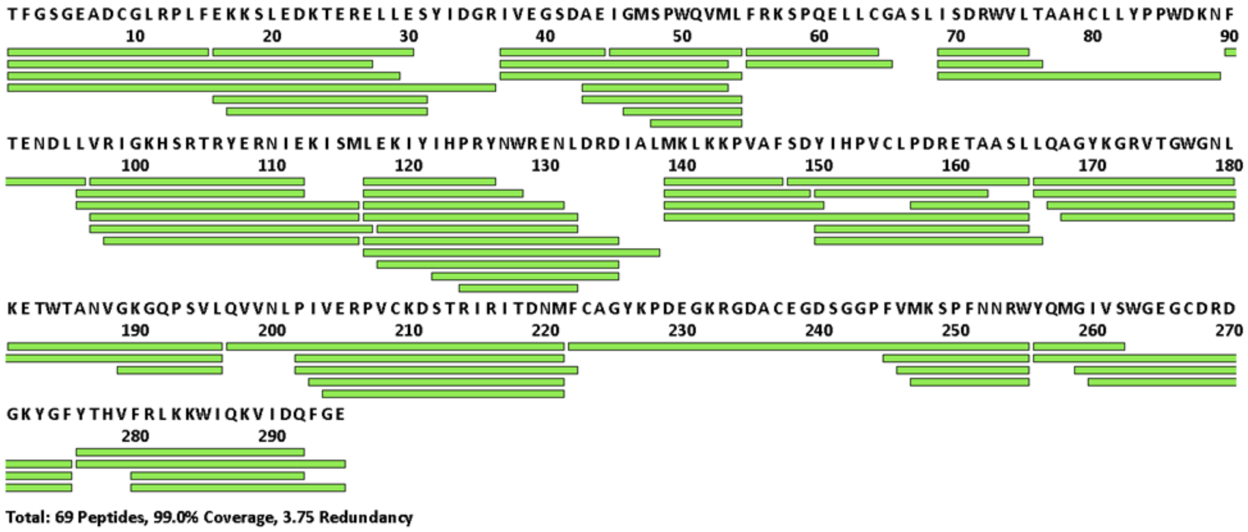

Fig. S3. Coverage map showing the peptides identified and evaluated during HDX-MS experiments on WT thrombin and thrombin-TM456.

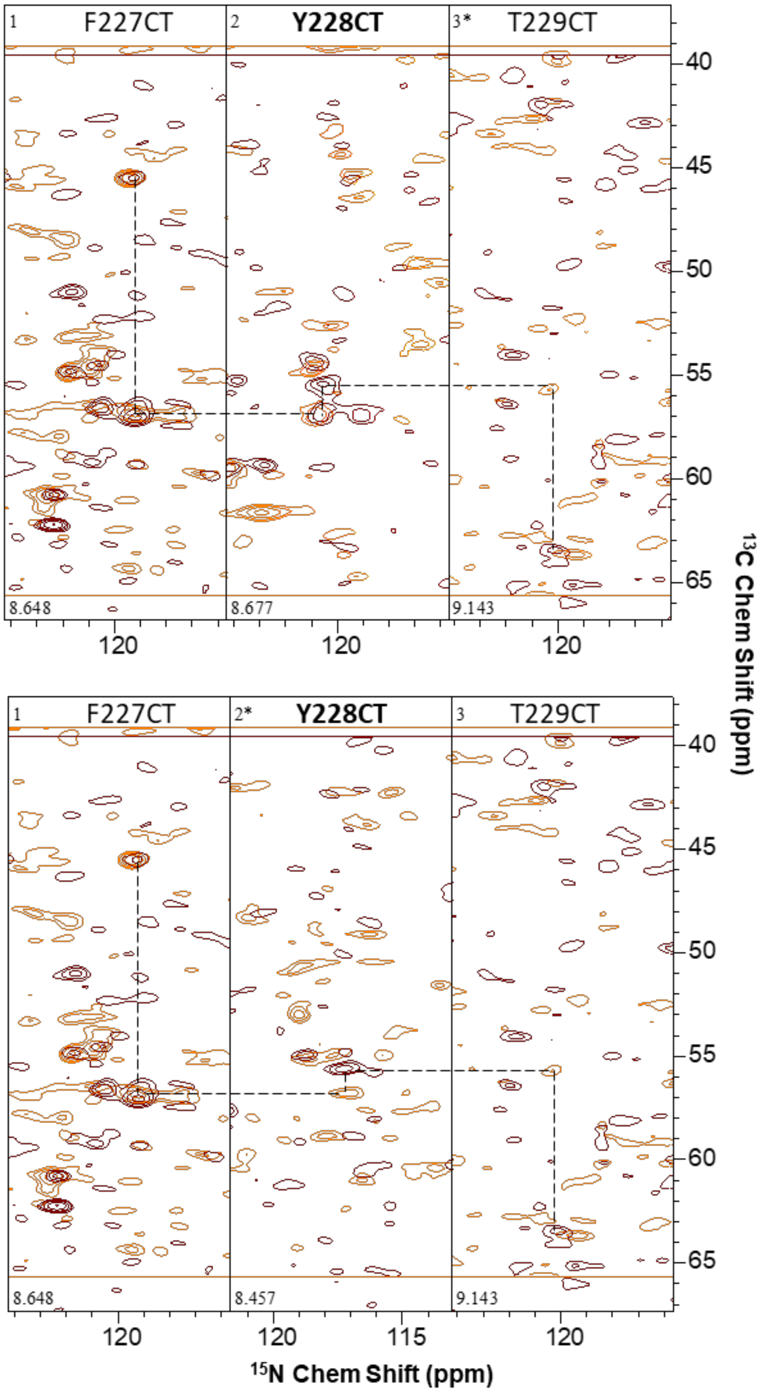

Fig. S4. Backbone walking from Ca_i to Ca_{-i} HNCA peaks for both residue 228 assignments. The brown and orange spectra correspond to the HNCA and HNcoCA spectra taken at 800 MHz for thrombin-TM456.

Table S1. Experimental details for 3D spectra collected for backbone assignments and 2D relaxation dispersion spectra.

Experiment	# Scans	Spectral Window (ppm) $^{1}H \times (^{13}C \times)^{15}N$	Complex Points $^{1}H \times (^{13}C \times)^{15}N$	Offset (ppm) $^{1}H \times (^{13}C \times)^{15}N$	NUS sampling rate (%)
3D HNCA-TROSY*	64	17.3×31.6×36.6	1024×58×35	4.77, 54.7, 118.1	38
3D HN(CO)CA-TROSY	80	20×26.5×36.2	1024×56×48	4.77, 52.4, 118.6	36
3D HNCO-TROSY	64	20×14.6×36.2	1024×44×44	4.77, 175.5, 118.6	38
2D ^{15}N-HSQC-TROSY*	16	17.3×36.6	1024×128	4.77, 118.1	N/A
2D ^{15}N-CPMG-TROSY	96	16.7×34.6	1024×128	4.77, 118.7	N/A
2D ^{15}N-CPMG-TROSY‡	80	16.3×34.6	1024×98	4.77, 118.1	N/A

[a]All spectra were collected with a cryogenic probe at 600, 800‡ and 900* MHz (^{1}H) at a temperature of 298 K.
